# Personality traits among major depressive disorder in southern Thailand: a hospital-based case–control study

**DOI:** 10.1186/s40359-023-01057-x

**Published:** 2023-01-24

**Authors:** Jarurin Pitanupong, Adchara Sa-i

**Affiliations:** Department of Psychiatry, Faculty of Medicine, Prince of Songkla University, Hat Yai, Songkhla 90110 Thailand

**Keywords:** Depression, Depressive residual symptoms, Patients, Personality

## Abstract

**Background:**

Residual depression can cause functional impairment. This study aimed to assess personality traits among individuals with depression, to compare the results with personality traits found in outpatients without psychiatric disorders recruited from general practitioner settings, and to study the association between personality traits and the age-onset of depression, duration of treatment, and the presence of depressive residual symptoms.

**Methods:**

A case–control study surveyed Thai individuals with depression and outpatients without psychiatric disorders recruited from general practitioner settings at an outpatient clinic of Songklanagarind hospital, in 2022. The questionnaires were: (1) demographic data, (2) the PHQ-9 Thai version, and (3) the International Personality Item Pool-NEO (IPIP-NEO) Thai version. The difference between personality traits and the assigned clinical group were analyzed using the Student’s t-test and the Wilcoxon rank sum test. A generalized linear model was performed to examine differences of personality traits between the assigned clinical group (case–control), and the presence of depressive residual symptoms. The association between personality traits and treatment profiles was assessed by using an analysis of the variance test and the Kruskal–Wallis test.

**Results:**

In regards to 73 individuals with depression in the case group, and 73 gender-and age-matched outpatients without psychiatric disorders recruited from general practitioner settings in the control group; 78.1% of cases and 82.2% of controls were female. Thirty-eight (52.1%) cases had depressive residual symptoms. Regarding the IPIP-NEO Thai version, there was a statistically significant difference in Neuroticism, Extraversion, and Conscientiousness domains between the case and the control groups. In comparison to the control group, the case group scored higher on the Neuroticism domain, and lower on the Extraversion and Conscientiousness domains. Every 0.18-point reduction in the Neuroticism score and every 0.09-point increment in regards to the Openness score were associated with a 1-year increment of age-onset of depression. This study found an association between a higher score of Neuroticism and a lower score of Conscientiousness with the presence of depressive residual symptoms.

**Conclusion:**

Different personality profiles were found between individuals with depression and outpatients without psychiatric disorders recruited from general practitioner settings. Individuals with depressive residual symptoms featured a higher score of Neuroticism and a lower score of Conscientiousness. A higher score of Neuroticism and a lower score of Openness were associated with age-onset of depression, but no personality traits were associated with treatment duration.

## Background

Major depressive disorder (MDD) is one of the most common psychiatric problems, which can create burdens and risks for harm in many areas of patients’ lives [1]. The global prevalence of MDD is 4.7% [2]. In the USA, the 12-month and lifetime prevalence according to MDD is 5.3% and 13.2%, respectively [3]. In Thailand, it is estimated that 1.5 million Thai people suffer from depression. Females have a higher prevalence compared with males at 2.9% and 1.7%, respectively [4]. Additionally, MDD creates a mortality risk for suicide in individuals with MDD that is 20-fold greater than in the general population [5]. Moreover, MDD is identified to be associated with a higher risk of cardiovascular death and stroke mortality [6]. It also causes a huge economic burden [7] and potentially contributes to decreased workplace productivity, including absenteeism, which can result in lower income or unemployment [8]. By disability-adjusted life-years (DALYs), 2004, MDD is put in third place in regards to the leading causes of burden of diseases worldwide [9]. In Thailand, in 2013, a prior study showed that MDD was the third leading cause of DALYs loss in Thai females and it was in the top twelve causes of disease burden in regards to Thai males [4].

While the goal of MDD treatment is the complete elimination of symptoms and a return to premorbid functioning, actual remission rates only range from 25.0 to 40.0% [10]. In Southern Thailand, a prior study from the Department of Psychiatry at the Faculty of Medicine, Prince of Songkla University, reported that despite receiving antidepressants for more than 12 weeks, 45.4% of individuals with MDD reported the presence of depressive residual symptoms including sleep problems, feeling down, lack of pleasure, and poor appetite [11]. Individuals with MDD aged 18 to 24 years had a higher rate of depressive residual symptoms than the older group, the adjusted odds ratio was 12.08 [11]. Moreover, the treatment resistance rate is also high up to 30.0% of the treated individuals with MDD [12].

Additionally, a prior study found evidence that several risk factors were associated to MDD, these included genetic influences, childhood maltreatment, premorbid psychosocial functioning, and personality [13]. The personality aspect was more interesting due to its modifiable property. Many studies had found an association between some personality traits with a diagnosis of MDD [14, 15], and with the onset, severity, and treatment outcome of MDD [16, 17]. Knowledge of any differences in the personality traits of non-depressive patients compared to the patients with MDD may help in the identification of at-risk population for MDD in the future. Additionally, knowing individual personality traits can provide clinicians with information about coping styles. This is relevant as some inappropriate coping styles may lead to MDD or the presence of depressive residual symptoms despite taking antidepressants [18–20].

Many studies found an important association between some personality traits and MDD, but none of these was concerning the Thai population. As there are different temperaments, cultures, and social environments, the association of personality traits and MDD findings may be different in the Thai population, this has not been investigated before. Therefore, the purpose of this cross-sectional case–control study was to assess personality traits among individuals with MDD, to compare the observed results with personality traits found in the sample of outpatients without psychiatric disorders recruited from general practitioner (GP) settings, and to study the association between personality traits and the age-onset of MDD, duration of treatment, and the presence of depressive residual symptoms among individuals with MDD.

## Methods

After being approved by the Ethics Committees of the Faculty of Medicine at the Prince of Songkla University (REC: 64-586-3-4), this case–control study was conducted at a psychiatric and general practitioner outpatient clinic at Songklanagarind Hospital, which is an 800-bed university hospital, serving as a tertiary referral center in southern Thailand. All individuals with MDD and outpatients without psychiatric disorders, who had an appointment and were followed up at psychiatric and GP outpatient clinics; from March to June 2022, were invited to participate in the study.

Individuals with a first episode of MDD, as diagnosed by psychiatrists, were selected in the medical register, as the case group, based on the following criteria: ICD-10 code; F33.0–F33.9; except F33.3, aged more than 20 years, acknowledging their diagnosis and received antidepressants for more than 12 weeks, good understanding and use of Thai language, agreeing to participate in the study, and completing all parts of the questionnaires. Meanwhile, those who had more than one psychiatric diagnosis or comorbidity, were unaware of their diagnosis, did not wish to participate or decided to withdraw from the study, and lacked the mental capacity to complete all of the questionnaires, were excluded.

The gender-and age-matched control group was divided into periods of 10 years each and contained the outpatients who were aged more than 20 years old, had no history of psychiatric disorder, had a screening test of a score of PHQ-9 with a range from 0 to 4 meaning that they had none or minimal depression [21], had no history of substance use or personality disorders or the presence of chronic medical conditions such as cancer, or non-communicable diseases with complications, had the capacity to read and complete the questionnaires, and were willing to complete the questionnaires.

The research assistant approached all of the eligible individuals with MDD and the outpatients without psychiatric disorders recruited from GP settings for recruitment and handed them an information sheet, which delineated the rationale for the study and the allotted time to complete the survey. All eligible participants had at least 15–20 min to consider whether to collaborate in the study or not. If they decided to, they were asked to sign an informed consent declaration in regards to the use of their information in the research questionnaire and gave their permission for a retrospective medical chart review. Furthermore, they were informed that their data would remain anonymous and that they could withdraw at any stage in regard to the administration of the questionnaire without giving any reasons; and with no impact on their treatment. Furthermore, participants willing to collaborate were invited to a private location to complete the questionnaires and if the participants exhibited a high level of distress or worry, advice and/or further clinical management was provided to them.

In regards to the sample calculation, a literature review found that the mean scores for the Neuroticism (N) personality trait in connection to the general population, individuals with MDD who gained remission, and people who still had symptoms despite proper treatment were 50.8 ± 10.6, 55.4 ± 9.0, and 60.9 ± 10.8, respectively. The mean score for Extraversion (E) personality traits in the same study groups were 53.3 ± 47.5, 47.5 ± 9.7, and 37.0 ± 11.2, respectively [22]. To see a significant difference in regards to the mean score of personality domains between the general population with MDD subjects, and the calculated sample size, by using the ‘n.for.2means’ command by R program, a minimum of 73 subjects for each of the groups were required: 73 cases and 73 controls, a total of 146 participants.

### Questionnaires

The questionnaire for the case group consisted of 3 parts; general demographic information, PHQ-9, and personality testing. For the control group, the questionnaire consisted of 2 parts; general demographic information, and personality testing.

1. General demographic information inquired around areas related to gender, age, marital status, religion, education, income, hometown, underlying disease, age-onset of depression, duration of treatment, type, and the last dose of antidepressants.
2. The Patient Health Questionnaire-9 (PHQ-9) Thai version, a self-rating questionnaire to evaluate depression consisted of 9 questions. The score of each question employed a 4-point rating scale; 0 (never); 1 (rarely); 2 (sometime); 3 (always). The total score ranged from 0 to 27; 0–4 (no or minimal depression); 5–9 (mild depression); 10–14 (moderate depression); 15–19 (moderately severe); 20–27 (severe depression). The questionnaire demonstrated internal consistency with a Cronbach’s alpha coefficient of 0.79, sensitivity of 0.53 and specificity of 0.98. The Thai version of the PHQ-9 had acceptable psychometric properties for the screening of major depression in general practice with a recommended cut-off score of nine or greater [21]. The PHQ-9 questionnaire demonstrated internal consistency with a Cronbach’s alpha coefficient of 0.92 for the data in this study.
3. International Personality Item Pool-NEO (IPIP-NEO) Thai version, a self-report questionnaire to evaluate personality in 5 domains. It consisted of 30 questions evaluating an individual’s behaviors; 8 items for Neuroticism (N); 3 items for Extraversion (E); 5 items for Agreeableness (A); 6 items for Openness (O); and 8 items for Conscientiousness (C). The score of each question employed a 5-point rating scale; 1 (disagree strongly or very inaccurate); 2 (disagree a little or moderately inaccurate); 3 (neither agree nor disagree or neither accurate nor inaccurate); 4 (agree a little or moderately accurate); 5 (strongly agree or very accurate). The questionnaire demonstrated internal consistency with a Cronbach’s alpha coefficient of N = 0.83, E = 0.76, O = 0.67, A = 0.37, C = 0.73 [23]. The IPIP-NEO questionnaire demonstrated internal consistency with a Cronbach’s alpha coefficient of 0.87 for the data in this study.

### Definition

Depressive residual symptoms among individuals with MDD were identified in the case of individuals with MDD, who received antidepressants more than 12 weeks ago [24, 25] and still had a PHQ-9 score of nine or greater [11, 21].

### Statistical analysis

Data distribution was analyzed using the Shapiro–Wilk test. Descriptive statistics were calculated using proportion, mean, standard deviation (S.D), median, and interquartile range (IQR) for patient demographic data. The comparison between groups was done using a Chi-square test, Wilcoxon rank sum test, and Student’s t-test. A generalized linear model was performed to examine differences of personality traits between the assigned clinical group (case–control), and depressive residual symptoms (PHQ-9 ≥ 9) in the case group. Additionally, the researcher tried to evaluate the association between personality traits and duration of treatment which was analyzed using an analysis of variance (ANOVA) and a Kruskal–Wallis test. All data analysis was performed using R, version 4.1.0 (R Foundation for Statistical Computing). The *p* value was considered statistically significant at the *p* < 0.05 threshold.

## Results

### Demographic characteristics

From March to June 2022, the participants in this study were 146 adults. Participants either met the criteria for the case study group (n = 73) or the control group (n = 73). In the case group, participants were of a median age (IQR) of 45 (33, 59) years, female (78.1%), Buddhist (84.7%), married (45.2%), completed bachelor’s degree or above (60.3%), and unemployed or student (44.4%). In the control group, participants were of a median age (IQR) of 47 (34, 58) years, female (82.2%), Buddhist (88.9%), married (52.1%), completed bachelor’s degree or above (74.0%), and working as employees in government agencies or state enterprises (55.6%).

Their general medical illness was hypertension (60.3%), dyslipidemia (47.8%), diabetes (28.4%), gastritis (9.6%), and diarrhea (4.1%). There was no other statistically significant difference in other demographic characteristics between the case and control groups except the occupation (Table 1).

**Table 1:** Demographic characteristics between outpatients without psychiatric disorders recruited from general practitioner settings and individuals with major depressive disorder

Demographic characteristics	Number (%)		Statistics	*p* value
	GP outpatients (n = 73)	MDD outpatients (n = 73)		
Gender			χ^2^ (1) = 0.17	0.678
Male	13 (17.8)	16 (21.9)		
Female	60 (82.2)	57 (78.1)		
Age (years)			Ranksum test	0.998
Median (IQR)	47 (34, 58)	45 (33, 59)		
Religion			χ^2^ (1) = 0.24	0.622
Buddhism	64 (88.9)	61 (84.7)		
Islam/Christianity/other	8 (11.1)	11 (15.3)		
Marital status			χ^2^ (1) = 0.44	0.508
Single/divorced	35 (47.9)	40 (54.8)		
Married	38 (52.1)	33 (45.2)		
Education level			χ^2^ (3) = 5.79	0.122
Secondary school or below	7 (9.6)	7 (9.6)		
High school	9 (12.3)	11 (15.1)		
Diploma	3 (4.1)	11 (15.1)		
Bachelor’s degree or above	54 (74)	44 (60.3)		
Occupation			χ^2^ (3) = 10.22	0.017^*^
Government employees officer/State enterprise officer/Private company employee	35 (55.6)	21 (29.2)		
Employee/agriculture	6 (9.5)	9 (12.5)		
Merchant/personal business	7 (11.1)	10 (13.9)		
Unemployed/student	15 (23.8)	32 (44.4)		

*Statistically significant difference between group

*IQR* Interquartile range

### Depressive symptom profiles and treatment in the case group

**Fig. 1:**
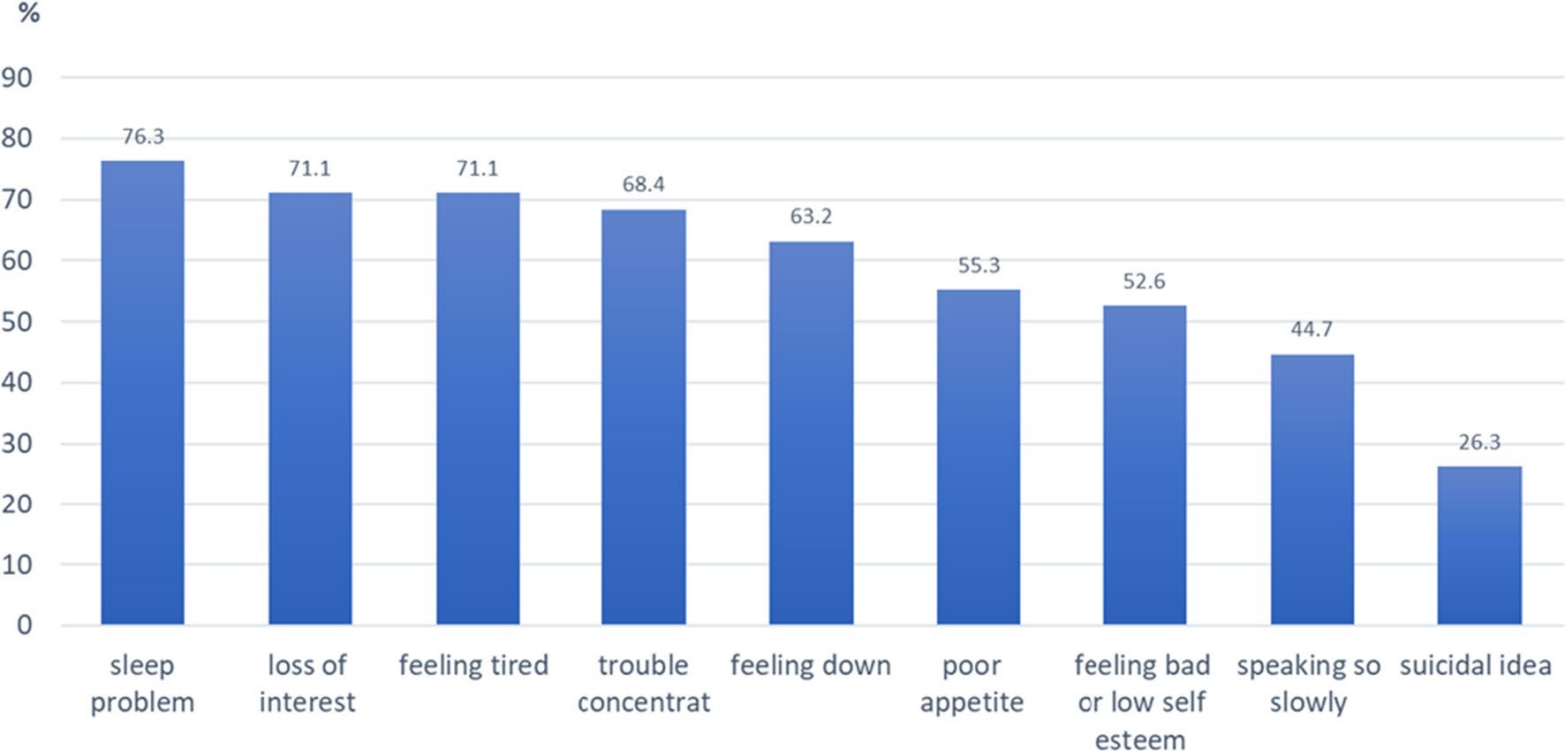
Frequency of depressive residual symptoms (n = 38)

Of all 73 cases, the mean ± S.D of age onset of MDD was 40.4 ± 15.9 years, and the median (IQR) duration of treatment was 24 (12, 48) months. Additionally, 38 cases (52.1%) had a PHQ-9 score ≥ 9. This meant that they still had symptoms of MDD or depressive residual symptoms [11, 21, 24, 25]. The depressive residual symptoms were insomnia (76.3%), loss of interest or pleasure (71.1%), feeling tired (71.1%), and trouble in concentration (68.4%) (Fig. 1). Concerning the type of antidepressants, the majority of cases (69.9%) received Selective Serotonin Reuptake Inhibitors (SSRIs) (Table 2).

**Table 2:** Treatment among individuals with major depressive disorder (N = 73)

Type of antidepressants	Number (%)
TCAs	10 (13.7)
Nortriptyline	5 (6.8)
Amitriptyline	3 (4.1)
Imipramine	2 (2.7)
Clomipramine	0
SSRIs	51 (69.9)
Sertraline	33 (45.2)
Fluoxetine	9 (12.3)
Fluvoxamine	5 (6.8)
Escitalopram	4 (5.5)
Others	36 (49.3)
Bupropion	13 (17.8)
Venlafaxine	10 (13.7)
Valdoxan	9 (12.3)
Mianserin	8 (11.0)
Vortioxetine	7 (9.6)
Mirtazapine	4 (5.5)
Duloxetine	1 (1.4)

### Personality profile of the case and control groups

In regards to the IPIP-NEO Thai version, there was a statistically significant difference in Neuroticism (*p* < 0.001), Extraversion (*p* < 0.001), and Conscientiousness (*p* < 0.001) between the case and control groups (Table 3). All 73 case groups scored higher in the Neuroticism domain by 8.10 (95% CI = 6.24, 9.95) compared to the control group. On the other hand, the case group scored lower in the Extraversion and Conscientiousness domains by − 1.25 (95% CI = − 2.07, − 0.42), and − 3.82 (95% CI = − 5.32, − 2.33), respectively, compared to the control group.

**Table 3:** Personality profile of outpatients without psychiatric disorders recruited from general practitioner settings and individuals with major depressive disorder

Personality traits	Number (%)		Statistics	*p* value
	GP outpatients (n = 73)	MDD outpatients (n = 73)		
Neuroticism			Ranksum test	< 0.001^*^
Median (IQR)	14 (12,17)	24 (17,27)		
Extraversion			Ranksum test	< 0.001*
Median (IQR)	10 (8,11)	9 (7,11)		
Agreeableness			Ranksum test	0.406
Median (IQR)	19 (18,21)	19 (17,21)		
Openness			t (144) = 0.07	0.942
Mean (SD)	21.3 (3.2)	21.3 (3.6)		
Conscientiousness			Ranksum test	< 0.001*
Median (IQR)	33 (30,35)	28 (25,33)		

*Statistically significant difference between group

*IQR* Interquartile range, *SD* Standard deviation

### Association of personality profile, age-onset of depression, duration of treatment, and presence of residual symptoms

Out of 73 individuals with MDD, there was a statistically significant association between Neuroticism and Openness scores with age-onset of MDD. Every 0.18-point reduction of Neuroticism scores was associated with the 1-year increment of age-onset of development of MDD (95% CI = − 0.28 to − 0.08, *p* < 0.001). On the other hand, every 0.09-point increment of the Openness score was associated with a 1-year increment of age-onset of MDD (95% CI = 0.14 to 0.04, *p* < 0.001).

According to the duration of treatment, the analyzed results did not show any statistically significant association between personality traits and duration of treatment (Table 4). However, there was an association between the presence of depressive residual symptoms and the personality traits of Neuroticism, and Conscientiousness (*p* < 0.001 and *p* = 0.002, respectively) (Table 5). The individuals with MDD who reported depressive residual symptoms had higher Neuroticism scores of 8.45 (95% CI = 5.81, 11.09) and lower Conscientiousness scores by − 3.82 (95% CI = − 6.17, − 1.46) than individuals with MDD who had no presence of depressive residual symptoms.

**Table 4:** Personality traits and duration of treatment among individuals with major depressive disorder (N = 73)

Personality traits	Number (%)			Statistics	*p* value
	≤ 2 years (n = 39)	> 2–5 years (n = 20)	> 5 years (n = 14)		
Neuroticism				F (2,70) = 2.93	0.060
Mean (SD)	24.2 (7.8)	22.4 (6.3)	19 (4.1)		
Extraversion				F (2,70) = 0.13	0.882
Mean (SD)	8.7 (2.6)	8.8 (2.7)	9.1 (3.3)		
Agreeableness				Kruskal–Wallis test	0.103
Median (IQR)	18 (17,20.5)	20 (18.8,22)	18 (15.2,19.8)		
Openness				F (2,70) = 2.05	0.136
Mean (SD)	20.9 (3.4)	22.7 (4.4)	20.5 (2.8)		
Conscientiousness				F (2,70) = 0.89	0.414
Mean (SD)	28 (5.4)	30 (6.0)	28.6 (4.1)		

*IQR* Interquartile range, *SD* Standard deviation

**Table 5:** Personality traits and the presence of depressive residual symptoms among individuals with major depressive disorder (N = 73)

Personality traits	Number (%)		Statistics	*p* value
	PHQ < 9 (n = 35)	PHQ ≥ 9 (n = 38)		
Neuroticism			t (71) = 6.38	< 0.001*
Mean (SD)	18.3 (5.1)	26.7 (6.1)		
Extraversion			t (71) = 1.43	0.157
Mean (SD)	9.3 (2.7)	8.4 (2.8)		
Agreeableness			Ranksum test	0.477
Median (IQR)	19 (16, 21)	18 (17,20)		
Openness			t (71) = 0.71	0.482
Mean (SD)	21.7 (3.6)	21.1 (3.7)		
Conscientiousness			t (71) = 3.22	0.002*
Mean (SD)	30.7 (5.0)	26.8 (5.1)		

*Statistically significant difference between group

Moreover, among individuals with MDD who reported depressive residual symptoms (38 cases), the participants who selected the “always” scale compared to the “rarely” scale had higher Neuroticism scores with a significant difference in these depressive residual symptoms domains including; loss of interest (*p* = 0.026), feeling down (*p* = 0.020), sleep problem (*p* = 0.021), feeling bad to self or low self-esteem (*p* < 0.001), trouble concentration (*p* < 0.001), and psychomotor retardation (*p* < 0.001). In contrast to the Conscientiousness domain, the participants who selected the “always” scale compared to the “rarely” scale had lower Conscientiousness scores with a significant difference in these depressive residual symptoms including; loss of interest (*p* = 0.013), feeling bad to self or low self-esteem (*p* = 0.025), trouble concentrating (*p* = 0.04).

## Discussion

This study indicated that there was a statistically significant difference in regard to the traits of Neuroticism, Extraversion, and Conscientiousness between the individuals with MDD and outpatients without psychiatric disorders recruited from GP settings. The individuals with MDD had a higher score of Neuroticism, but a lower score of Extraversion, and Conscientiousness. Additionally, individuals with MDD who had depressive residual symptoms scored higher on Neuroticism and lower on Conscientiousness than the individuals with MDD who had not.

In regards to this study’s result, we found differences between personality traits among Thai individuals with MDD and outpatients without psychiatric disorders recruited from GP settings, especially in connection to the traits of Neuroticism, Extraversion, and Conscientiousness. The result was similar to those results reported by a prior meta-analysis, including 175 studies published from 1980 to 2007 [14] as well as to that of another study in the Netherlands [16], and the USA [17]; which also suggested that the presence of MDD was significantly associated with higher Neuroticism, and lower Extraversion and Conscientiousness [16, 17]. Trying to identify the association between personality traits and the age-onset of MDD, our study found that a reduction of the Neuroticism score and increments of the Openness score was associated with increments of the age-onset of MDD. These findings were opposite to the aforementioned study from the Netherlands, which found that the earlier onset of MDD was significantly associated with a higher Openness score [16]. A possible explanation for this discrepancy could be that there were age range differences, in our study their median age was 45 years while in the previous study, the participants were older adults.

An investigation study in regards to any causal relationship between the trait of Neuroticism and MDD via Mendelian Randomization (MR)identified strong evidence that the trait of Neuroticism was a causal risk factor for MDD, and every 1-point reduction in the Neuroticism score reduced the log odds of MDD by 0.25 [26]. Furthermore, a reduction of the Neuroticism score by 4 points reduced the chance of MDD by about 25.0% [26]. However, due to our cross-sectional study design, our result cannot support a causal relationship. Asides from looking at personality traits as risk factors for MDD, depression itself may have had a state effect due to its characteristic patterns of thinking, feeling, and behaving in a concrete situation at a specific moment in time, on personality scoring. Many prior studies found that individuals with MDD reported a higher score of Neuroticism when they were depressed than when they were not. Even though the scores changed after remission, the personality traits and characteristics tended to be well preserved [27, 28]. However, the state effect on personality assessment was worth noting.

According to depressive residual symptoms, our study identified that individuals with MDD who reported depressive residual symptoms had a higher score of Neuroticism and lower score of Conscientiousness than individuals with MDD who had no presence of depressive residual symptoms. This result is also consistent with a prior study in connection to a longitudinal analysis, on the improvement of older adults with MDD who received treatment for 3 and 12 months, which found a positive correlation between improvement and lower Neuroticism scores [17]. In another study, higher scores on the Openness domain at the beginning of treatment were associated with lower MDD severity at treatment completion [29]. Our study did not find any significant association between depressive residual symptoms and Openness scores, however, due to the cross-sectional study design, we cannot assess this point of information directly. Previously, only a few studies assessed the association between personality traits and depressive residual symptoms. One study found that remitted patients with residual nightmares scored higher on Neuroticism [30]. However, our result added some new knowledge to this area, as we found that treated individuals with MDD who had higher Neuroticism scores reported more frequent depressive residual symptoms such as the loss of interest, feeling down, sleep problems, feeling bad about self or low self-esteem, trouble concentration, and psychomotor retardation. Furthermore, treated individuals with MDD who had lower Conscientiousness scores reported more frequent depressive residual symptoms of loss of interest, feeling bad about self or low self-esteem, and trouble concentrating.

Finally, in the clinical field, knowing the personality traits of an individual with MDD potentially gave useful information to clinicians. The trait of Neuroticism was highly related to escape-avoidant coping interactions with life stressors that were related to developing MDD symptoms [18, 19]. Low Conscientiousness was related to a lack of problem-solving [20], which was found in MDD with the presence of depressive residual symptoms, as per our study. This was due to problematic coping, still interacting with everyday life stressors, and causing distress. Therefore, considering that personality traits might play a role in determining the choices of treatment for MDD. Individuals with MDD who had higher scores on Neuroticism were more likely to achieve remission by being treated with pharmacotherapy versus cognitive-behavioral therapy [29]. Individuals with MDD who reported depressive residual symptoms should be assessed in connection to their personality traits to aid in determining whether to provide additional pharmacotherapy or psychotherapy, and to more effectively target improper coping styles. Furthermore, knowing the personality traits of non-depressive individuals might help identify people who were potentially more prone to develop MDD in the future, and provide early preventative interventions.

This study had a few noteworthy strengths and limitations. To our knowledge, this was the first case–control study investigating the association of personality traits and MDD in Thailand. However, this study provided limited results and scope of interpretation due to its cross-sectional design, lack of a baseline measurement and long-term follow-up, as well as the utilization of self-administered questionnaires, with the possibility of misunderstandings regarding the intended meaning of the questions. Nevertheless, to minimize this, questionnaires with good reliability were utilized (good Cronbach’s alpha coefficient values). Other drawbacks were that our data were quantitative, and the sample size was limited to the patients enrolled at only one hospital. Hence, this dataset might not fairly represent Thai individuals with MDD countrywide. Additionally, there was a difference in occupation between groups, and the control group consisted of the outpatients without psychiatric disorders who were selected from a clinical setting. They were not part of the general population. This may have affected the relevance of this study’s results to the general population. Also, we were concerned about any transcultural effects on personality traits and MDD characteristics, but our observed results were largely congruent with prior studies abroad.

It is recommended that future studies include all individuals with MDD, from all regions of Thailand. In other words, a comprehensive multi-center study should be conducted. Moreover, future research should concentrate on different instruments, utilize more qualitative designs, and employ longitudinal surveillance or long-term follow-ups.

## Conclusion

There were differences in the trait of Neuroticism, Extraversion, and Conscientiousness between individuals with MDD and outpatients without psychiatric disorders recruited from GP settings. Individuals with MDD had a higher score of Neuroticism and lower score of Extraversion and Conscientiousness than the other group. A lower score of Neuroticism and a higher score of Openness were associated with an increasing age-onset of MDD development. Individuals with MDD who reported depressive residual symptoms had a higher score of Neuroticism and lower score of Conscientiousness than individuals with MDD who had no presence of depressive residual symptoms. Therefore, clinicians may benefit from knowing patients’ personality traits as this could potentially help identify coping mechanisms, inform choices of treatment modality among individuals with MDD, and predict depressive residual symptoms. Furthermore, it is suggested that this could also assist in identifying individuals at a higher risk of developing MDD in the future.

## Data Availability

Data are available upon reasonable request. De-identified data are available upon request from the correspondent author.

## Abbreviations

IPIP-NEO: International Personality Item Pool-NEO
MDD: Major depressive disorder
PHQ-9: Patient Health Questionnaire-9
SSRIs: Selective serotonin reuptake inhibitors
